# Transient filament occlusion of the middle cerebral artery in rats: does the reperfusion method matter 24 hours after perfusion?

**DOI:** 10.1186/1471-2202-13-154

**Published:** 2012-12-29

**Authors:** Jian-Ren Liu, Ulf R Jensen-Kondering, Jia-Jun Zhou, Fen Sun, Xiao-Yan Feng, Xiao-Lei Shen, Günther Deuschl, Olav Jansen, Thomas Herdegen, Johannes Meyne, Yi Zhao, Christoph Eschenfelder

**Affiliations:** 1 Department of Neurology, University of Schleswig-Holstein, Campus Kiel, Christian-Albrechts-University Kiel, Kiel, Germany; 2 Institute of Neuroradiology, University of Schleswig-Holstein, Campus Kiel, Christian-Albrechts-University Kiel, Kiel, Germany; 3 Department of Neurology, Shanghai Ninth People's Hospital Affiliated to Shanghai Jiao Tong University School of Medicine, 639 Zhizaoju Road, Huangpu District, Shanghai, China; 4 Institute of Pharmacology, University of Schleswig-Holstein, Campus Kiel, Christian-Albrechts-University Kiel, Kiel, Germany; 5 Department of Neurology, Second Affiliated Hospital, Zhejiang University School of Medicine, Hangzhou, China; 6 Boehringer Ingelheim Pharma GmbH & Co. KG, Ingelheim am Rhein, Germany

**Keywords:** Middle cerebral artery occlusion, Ischemia, Reperfusion method, Bilateral Carotid reperfusion, Unilateral Carotid reperfusion, Neurological deficits, Diffusion weighted imaging, Perfusion weighted imaging, Partial oxygen pressure

## Abstract

**Background:**

There are two widely used transient middle cerebral artery occlusion (MCAO) methods, which differ in the use of unilateral or bilateral carotid artery reperfusion (UNICAR and BICAR). Of the two methods, UNICAR is easier to perform. This study was designed to comprehensively compare the two reperfusion methods to determine if there are any differences in outcomes.

**Results:**

The UNICAR and BICAR groups each included 9 rats**.** At baseline, the average pO_2_ was 20.54 ± 9.35 and 26.43 ± 7.39, for the UNICAR and BICAR groups, respectively (*P* = 0.519). Changes in pO_2_, as well as other physiological parameters measured within the ischemic lesion, were similar between the UNICAR and BICAR groups during 90 min of MCAO and the first 30 min of reperfusion (all *P* > 0.05). Furthermore, both the Bederson score and Garcia score, which are used for neurological assessment, were also similar (both *P* > 0.05). There were also no significant differences in T2WI lesion volume, DWI lesion volume, PWI lesion volume, or TTC staining infarct volume between the two groups (all *P* > 0.05).

**Conclusion:**

UNICAR and BICAR have similar capability for inducing acute brain ischemic injury and can be considered interchangeable up to 24 hours after reperfusion.

## Background

The transient middle cerebral artery occlusion (MCAO) stroke model has been widely used to study the effects of cerebral ischemia (for review see [1]). Furthermore, there have been many studies on transient MCAO, demonstrating that rat strain [2], animal age [3], ischemia duration [4], and suture type [5] contribute to variability of outcomes. However, we are unaware of any research comparing differences in reperfusion techniques.

There are two basic reperfusion methods (return of blood flow) in models of transient MCAO. In the bilateral carotid artery reperfusion (BICAR) method, the common carotid artery (CCA) is temporarily occluded; a filament is put through an opening cut in the external carotid artery (ECA) and then pushed through the internal carotid artery (ICA) to occlude the middle cerebral artery (MCA). Prior to reperfusion, the filament is pulled out from the ECA, which remains occluded while the CCA and ICA are reopened. This method results in the brain being reperfused by both carotid arteries. In the unilateral carotid artery reperfusion (UNICAR) method, the CCA and ECA are permanently ligated and the filament is pushed through the opening of the CCA distal to the ligation point and then threaded up into the ICA to occlude the MCA. Before reperfusion, the filament is withdrawn while the CCA and ECA are still occluded; as a result, only the carotid artery contralateral to the ischemic hemisphere reperfuses the brain.

The primary difference between the UNICAR and BICAR methods is the complexity of the procedure. In our experience, UNICAR is easier to perform since it does not require separating the ECA prior to MCAO, reopening the CCA, and ligating the nub of the ECA before inducing reperfusion. This increases the possibility of damaging the soft tissues and cranial nerves leading to difficulty in food and water intake, which can confound the results of the study. Furthermore, as the CCA is thicker and straighter than the ECA, it is easier to cut as well as insert the suture.

We hypothesize that BICAR results in more abundant blood supply to the ischemic brain tissue, thereby leading to better locomotor and histological outcomes than UNICAR. To test this hypothesis we compared both reperfusion methods in regards to cerebral blood flow (CBF) and partial oxygen pressure (pO_2_) within the ischemic brain tissue during MCAO/reperfusion. We also studied infarct volume at 24 h after reperfusion by using comprehensive modalities including histological assessment and magnetic resonance imaging (MRI) including T2-weighted MRI (T2WI), diffusion-weighted MRI (DWI) and perfusion-weighted MRI (PWI). In addition neurological deficits were analyzed using two well-established neurological grading systems.

## Methods

### Transient MCAO models

**Figure 1:**
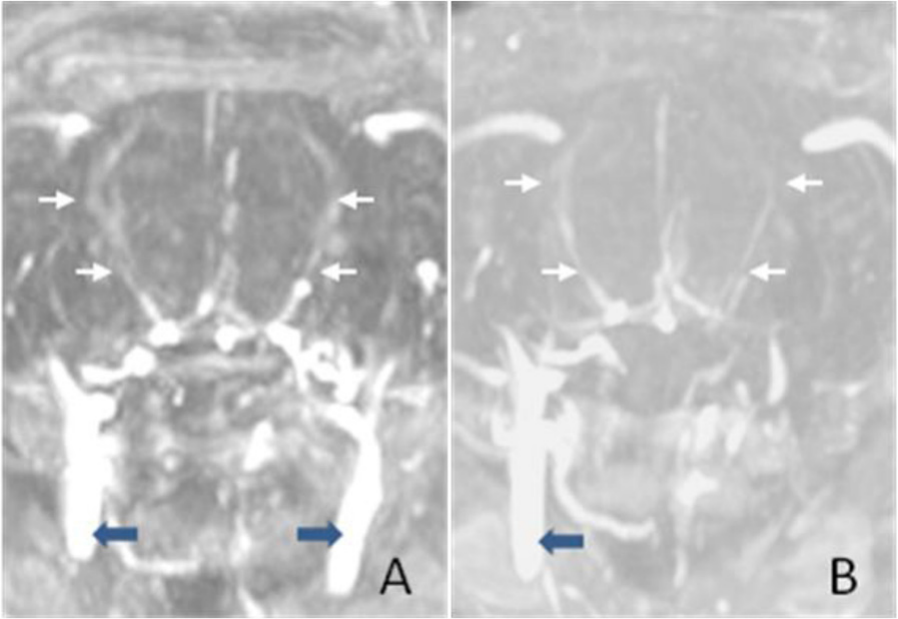
**Magnetic resonance angiography in two different intraluminal middle cerebral artery occlusion (MCAO)/reperfusion rat models. A**, UNICAR group, only the right common carotid artery supplied the brain (thick arrows) after reperfusion, the left common carotid artery remained occluded after reperfusion, the left brain is supplied by the contralateral carotid artery via the communication artery; **B**, BICAR group, bilateral common carotid artery (thick arrows) supplied the brain after reperfusion. The bilateral middle cerebral arteries after reperfusion are shown well in either UNICAR or BICAR model, which indicates that the two models have similar extent of reperfusion. The thin arrows indicate middle cerebral artery in **A** and **B.**

All animal experiments were performed according to the German laws for animal protection and the National Institutes of Health guidelines for the use and care of laboratory animals (all experiments were approved by the Ministerium fuer Landwirtschaft, Umwelt und Ländliche Räume des Landes Schleswig-Holstein, Kiel, Germany). Male Sprague–Dawley rats (Charles River, Germany) weighing 200–250 g and aged 6–8 weeks were provided with food and water *ad libitum* and housed in a 12:12-h light/dark cycle at 24°C with appropriate humidity. Rats were fasted for one night before the MCAO procedure. Body temperature was maintained at 37.0 ± 0.5°C with a heating pad during all procedures. Chloral hydrate (400 mg/kg body weight) injected intraperitoneally was used as anesthetic for all surgical procedures [6]. The rats were randomized to the UNICAR group (n = 15) and BICAR group (n = 15). For the UNICAR group, an intraluminal MCA occlusion method, combined with unilateral (contralateral) carotid reperfusion was used. Under general anesthesia, the right ECA and CCA were ligated and then the right MCA was occluded for 90 min by means of a silicon-coated 4–0 nylon filament introduced via the ICA through a cut in the right CCA 0.5 cm proximal to the carotid bifurcation. Reperfusion was achieved by pulling out the monofilament from the CCA [7-11] (Figure 1A). For the BICAR group, the right CCA was temporally occluded after anesthesia. The right ECA was separated and occluded permanently at its distal part, the right MCA was occluded for 90 min with a silicon-coated 4–0 nylon filament, introduced through an opening cut in the ECA and then advanced through the ICA. After withdrawing the filament for reperfusion, the ECA was occluded permanently while the CCA was reopened as previously described [12,13] (Figure 1B). 

Inclusion criteria required each animal to exhibit a decrease in pO_2_ of > 50% within the infarct area (determined as described below) during MCAO and to survive for at least 24 hours following MCAO.

There were no deaths prior to randomization. In the UNICAR group, one rat died of excessive anesthesia and four rats were excluded from analysis because their pO_2_ did not meet the criteria. In addition, one rat died of cerebral ischemia after MCAO. The other 9 rats were included in the analysis. In the BICAR group, 2 rats died of subarachnoid hemorrhage and one died of excessive anesthesia. Also, the pO_2_ changes of 3 rats did not meet the inclusion criteria and they were excluded. The remaining 9 rats were included in the analysis. The analysis therefore included 9 rats in each group.

### Measurements

#### Detection of pO_2_ in ischemic brain tissue

**Figure 2:**
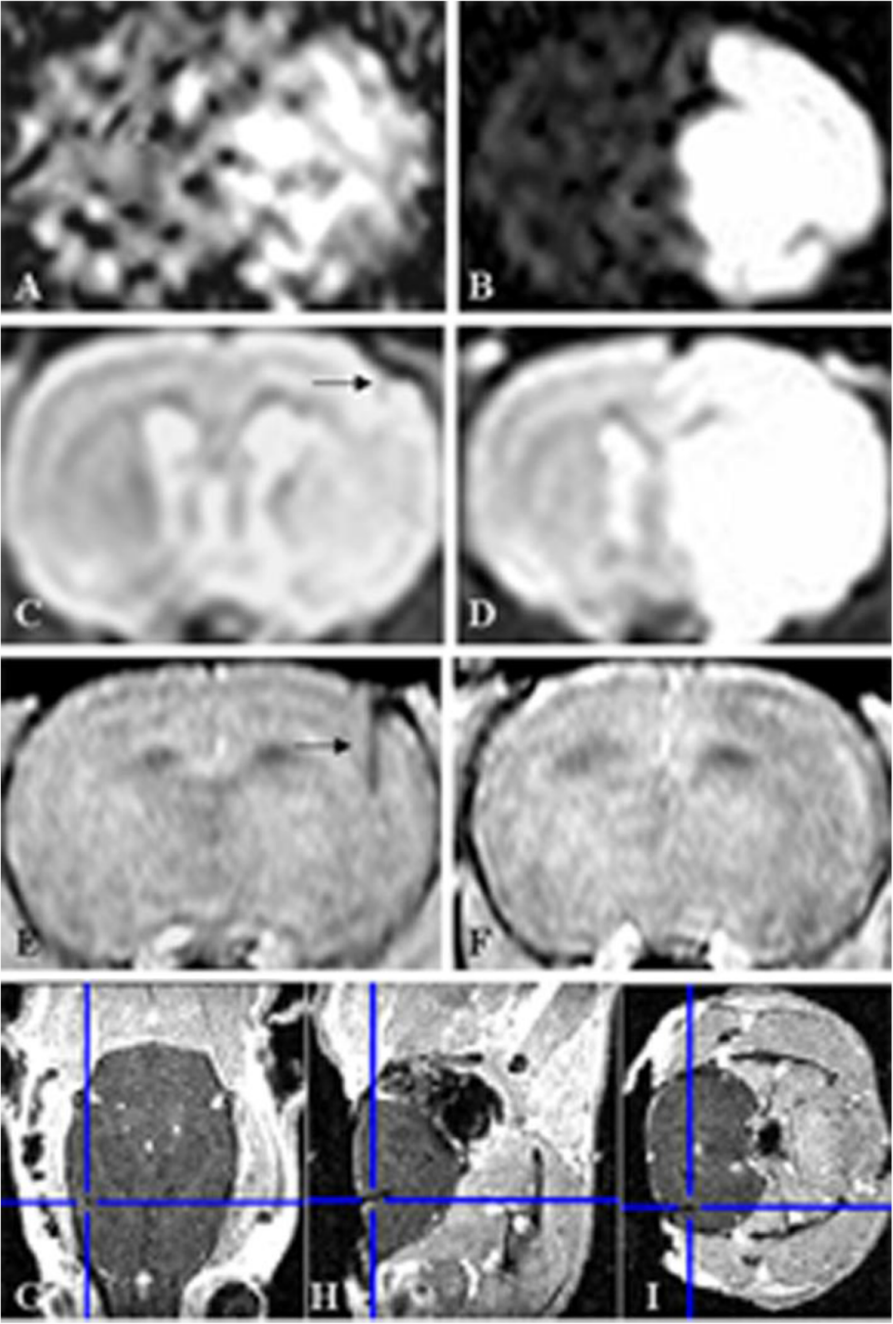
**Magnetic resonance imaging showing localization of the pO_**2 **_detecting probe (Licox® CMP) and infarct lesion. A**, **C**, **E**, DWI, T2WI, and time-of-flight (TOF) image after 90-min ischemia and 1-h reperfusion. **B**, **D**, **F**, DWI, T2WI, and TOF image after 90-min ischemia and 24-h reperfusion. **G**, **H**, **I**, three dimensional localization of the probe. DWI, diffusion-weighted images; T2WI, T2-weighted images.

Prior to either MCAO procedure a pO_2_ detecting probe (Licox® CMP, Kiel, Germany) was implanted according to previously reported stereotactic coordinates [14] to detect pO_2_ in the core of the ischemic brain tissue during MCAO/reperfusion as shown in Figure 2. 

#### MRI scan

Three days before the MCAO procedure, a venous catheter was prepared for intravenous contrast injection by inserting a polyethylene catheter (PE-50) into the inferior vena cava. The catheter was filled with heparinized saline, passed through a subcutaneous tunnel, sealed, and then secured at the back of the neck to prevent the animal from removing or damaging it. MRI was performed on anesthetized rats placed in a supine position at 1 hr (DWI and PWI) and 24 hrs (T2WI, DWI, and PWI) following reperfusion with a 3.0 T Philips scanner using a 3 T Solenoid Rat Coil (PFL-Hamburg, Germany). The scanning parameters used are listed in Table 1. Contrast injection was performed as previously described [15]. A neuroradiologist blinded to the type of surgery. 

**Table 1:** Parameters of magnetic resonance imaging

	**T2WI**	**DWI**	**PWI**
TE (ms)	5860	2399	12
TR (ms)	100	165	24
FOV (mm)	64 × 64	50 × 50	64 × 48
Matrix	128 × 126	96 × 77	64 × 45
Slice thickness (mm)	0.7	1.3	1.5
Gap (mm)	0.2	0.2	-
b-values	-	0 and 2000	-
Dynamics	-	-	40

TE, echo time; TR, repetition time; FOV, field of view; T2WI, T2-weighted images; DWI, diffusion-weighted images; PWI, perfusion-weighted images.

On DWI, infarcted brain tissue was defined on a Philips ViewForum workstation (Philips, The Netherlands) using an apparent diffusion coefficient (ADC) threshold of 0.53 × 10-3 mm^2^/s, as described elsewhere [16]. On PWI, perfusion was calculated using the NordicIce package (NordicIce, Norway) to determine the PWI lesion defined as tissue with a TTP > 7 sec [17,18].

Accurate calculation of infarct volume must compensate for the effect of brain edema. Thus, the corrected infarct area was calculated as that total area of the infarct area - (the ischemic hemisphere area - the contralateral hemisphere area), as previously described [19]. The edema area was calculated as ischemic hemisphere area - contralateral hemisphere area. Corrected infarct volume was then calculated by the sum of the corrected infarct area on each section multiplied by slice thickness.

MRI images of insufficient quality were excluded from analysis. T2WI images of one rat were excluded; DWI and PWI images of 4 and of 3 rats respectively were excluded.

#### Evaluation of neurological deficits: the Bederson and Garcia score

Two neurological grading systems were used to assess the effects of ischemia. The first grading system was published by Bederson et al. in 1986 [20]. This system consists of a scale from 0 to 3: (0) no observable deficit; (1) decreased forelimb resistance to a lateral push; (2) forelimb flexion; (3) circling behavior in addition to the former symptoms. The second system was introduced by Garcia et al. in 1995 [21]. It consists of 6 different criteria (spontaneous activity, symmetry in the movement of the 4 limbs, forepaw outstretching, climbing, body proprioception, and response to vibrissae touch). The individual performance in each test was rated on a 0 to 3 point subscore. The sum of all 6 individual subscores was then calculated to give a range of 3 – 18. Thus, the score in healthy rats would be 18. Two blinded to the type of surgery. The neurological scores of each rat were assessed the neurological score at 24 h following reperfusion as previously described [6,22].

#### Histological assessment of infarct volume by 2, 3, 5-triphenyl tetrazolium chloride (TTC) staining

TTC staining was used for histological infarct detection [23]. At 24 h after reperfusion, animals were deeply anesthetized with chloral hydrate and perfused intracardially as previously described [6,22]. The 24 h time point was chosen as it has been used previously in studies assessing pathophysiological mechanisms of cerebral ischemia [6,24,25]. Brains were removed, and 6 coronal sections (2 mm thick slices from anterior, 3.5 mm to anterior, 13.5 mm from bregma) were taken. Sections were then stained with 2% TTC and fixed by immersion in 10% phosphate-buffered paraformaldehyde. The TTC-stained brain sections were photographed and the infarct volumes calculated by an investigator blinded to the type of surgery. The infarct volume was then quantified with the use of imaging software (Image J, National institute of Health, USA) and with the same method as MRI scan see above).

#### Physiological evaluation

In addition to the experimental rats receiving the examinations mentioned above, MCAO was also performed on an additional 17 rats (8 in the UNICAR group and 9 in the BICAR group) in order to evaluate the physiological changes including changes in serum hemoglobin, serum pH, partial carbon dioxide pressure (pCO_2_), pO_2_, electrolytes, serum glucose, and mean arterial blood pressure. These rats were used only for physiological parameter analysis, since blood sampling and femoral catheterization could affect outcome of MCAO/reperfusion. Chloral hydrate (400 mg/kg body weight) injected intraperitoneally was used as the anesthetic for all surgical procedures. After anesthesia, a polyethylene catheter (PE-50) was inserted into the right femoral artery and connected with the Pressure Monitor BP1 to continuously monitor the mean arterial blood pressure (MABP). The physiological evaluations were performed at three time points: before MCAO, at 30 min after MCAO, and at 30 min after reperfusion.

### Statistical analysis

Quantitative data were expressed as mean ± standard deviation. Statistically significant differences in continuous variables (physiological parameters, DWI lesion volume, PWI lesion volume, infarct volume, edema volume, corrected infarct volume and neurological scores) between the UNICAR and BICAR groups were determined by independent t-test. The pO_2_ changes were compared between 2 groups at each time-point using a linear mixed model. The statistical analyses were performed using SAS software version 9.2 (SAS Institute Inc., Cary, NC, USA) and a two-tailed *P* < 0.05 indicated statistical significance.

## Results

Of the 18 rats (9 in each group), 17 (9 in the UNICAR group and 8 in the BICAR group) underwent T2WI at 24 hours, 14 rats (6 in UNICAR group and 8 in BICAR group) had DWI scan at 60 min and 24 hours after reperfusion, and 15 rats (7 in UNICAR group and 8 in BICAR group) had PWI scan at 60 min and 24 hours after reperfusion.

In the additional 17 rats used to investigate physiological parameters at 3 different time points (before MCAO, at 30 min after MCAO, and at 30 min after reperfusion). at all 3 time points, we found no significant differences between the UNICAR and BICAR groups for any measure (Table 2). 

**Table 2:** Comparison of physiological changes between the UNICAR and BICAR groups

	**UNICAR**	**BICAR**	***P*-value†**
	**(n = 8)**	**(n = 9)**	
Pre-MCAO			
Hgb (g/dl)	14.09 ± 1.03	13.62 ± 0.96	0.351
pH	7.37 ± 0.04	7.35 ± 0.04	0.252
pCO_2_ (mmHg)	43.38 ± 3.98	46.53 ± 4.51	0.149
pO_2_ (mmHg)	87.28 ± 6.07	89.00 ± 5.17	0.536
K^+^ (mmol/L)	3.86 ± 0.62	4.03 ± 0.39	0.499
Na^+^ (mmol/L)	140.24 ± 2.64	121.17 ± 43.80	0.228
Cl^-^ (mmol/L)	104.33 ± 3.66	101.62 ± 3.22	0.126
Glucose (mg/L)	139.75 ± 10.59	133.78 ± 6.50	0.176
MABP (mmHg)	86.25 ± 10.65	82.00 ± 6.48	0.330
MCAO 30’			
Hgb (g/dl)	13.99 ± 1.50	13.00 ± 1.48	0.193
pH	7.36 ± 0.03	7.35 ± 0.04	0.653
pCO_2_ (mmHg)	45.45 ± 5.49	47.74 ± 8.63	0.529
pO_2_ (mmHg)	83.33 ± 10.56	85.89 ± 3.47	0.529
K^+^ (mmol/L)	3.94 ± 0.43	4.10 ± 0.34	0.384
Na^+^ (mmol/L)	138.60 ± 2.84	135.78 ± 3.10	0.071
Cl^-^ (mmol/L)	106.39 ± 4.03	104.13 ± 3.65	0.245
Glucose (mg/L)	134.50 ± 11.20	132.56 ± 5.05	0.662
MABP (mmHg)	87.75 ± 7.78	81.78 ± 6.76	0.111
Rep 30’			
Hgb (g/dl)	12.84 ± 1.37	12.82 ± 1.29	0.981
pH	7.36 ± 0.03	7.33 ± 0.04	0.117
pCO_2_ (mmHg)	42.46 ± 8.58	44.34 ± 5.85	0.601
pO_2_ (mmHg)	96.36 ± 14.10	88.84 ± 6.17	0.195
K^+^ (mmol/L)	3.86 ± 0.57	3.79 ± 0.42	0.793
Na^+^ (mmol/L)	139.23 ± 2.53	136.41 ± 3.83	0.099
Cl^-^ (mmol/L)	106.00 ± 3.25	104.56 ± 4.36	0.456
Glucose (mg/L)	142.13 ± 17.69	133.56 ± 5.75	0.225
MABP (mmHg)	86.00 ± 10.89	81.11 ± 4.94	0.271

Data are presented as mean ± standard deviation.

Pre-MCAO: before MCAO; MCAO 30’: at 30 min after MCAO; Rep 30’: at 30 min after reperfusion. UNICAR, unilateral carotid artery reperfusion; BICAR, bilateral carotid artery reperfusion; Hgb, hemoglobin; MCAO, middle cerebral artery occlusion; MABP, mean arterial blood pressure.

† determined by independent t-test.

### Comparison of pO_2_ change during MCAO/reperfusion

**Figure 3:**
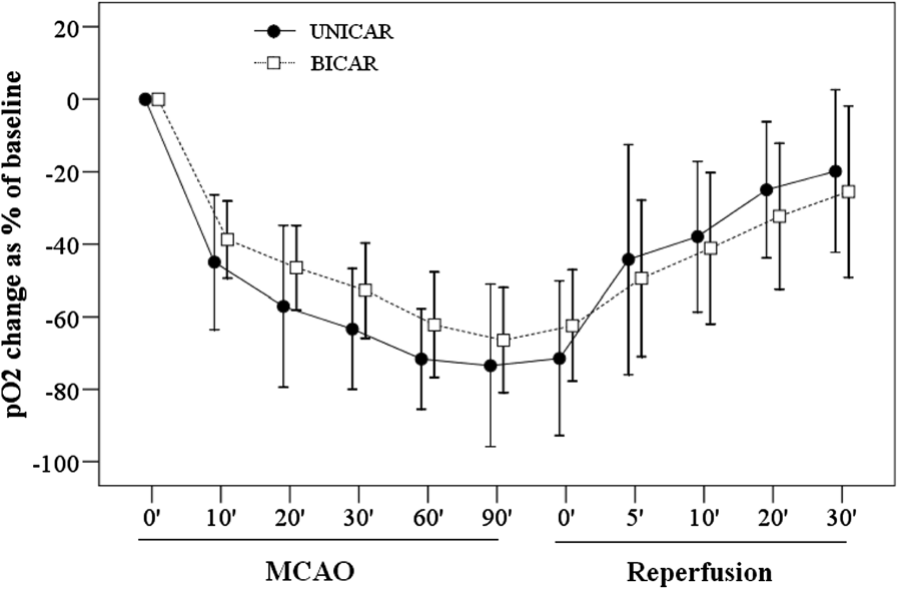
**Comparison of pO_**2 **_change between the unilateral (n = 9) and bilateral carotid artery reperfusion (n = 9) groups during middle cerebral artery occlusion/reperfusion. **pO_2_ change (%) =100 × (pO_2_ at each time point - baseline pO_2_)/ baseline pO_2_. MCAO, middle cerebral artery occlusion; UNICAR, unilateral carotid artery reperfusion; BICAR, bilateral carotid artery reperfusion.

At baseline, the average pO_2_ was 20.54 ± 9.35 and 26.43 ± 7.39, for UNICAR and BICAR groups, respectively (*P* = 0.519). The pO_2_ change percentages (compared to baseline) during MCAO (90 min) and reperfusion (30 min) are shown in Figure 3. For both groups, pO_2_ change percentages showed a decreasing trend over time during MCAO and an increasing trend over time during reperfusion. However, the pO_2_ change percentages between the UNICAR and BICAR groups did not differ significantly at any time point during MCAO or reperfusion (all *P* > 0.05). 

### Comparison of infarct volume, edema volume and corrected infarct volume on T2WI

**Figure 4:**
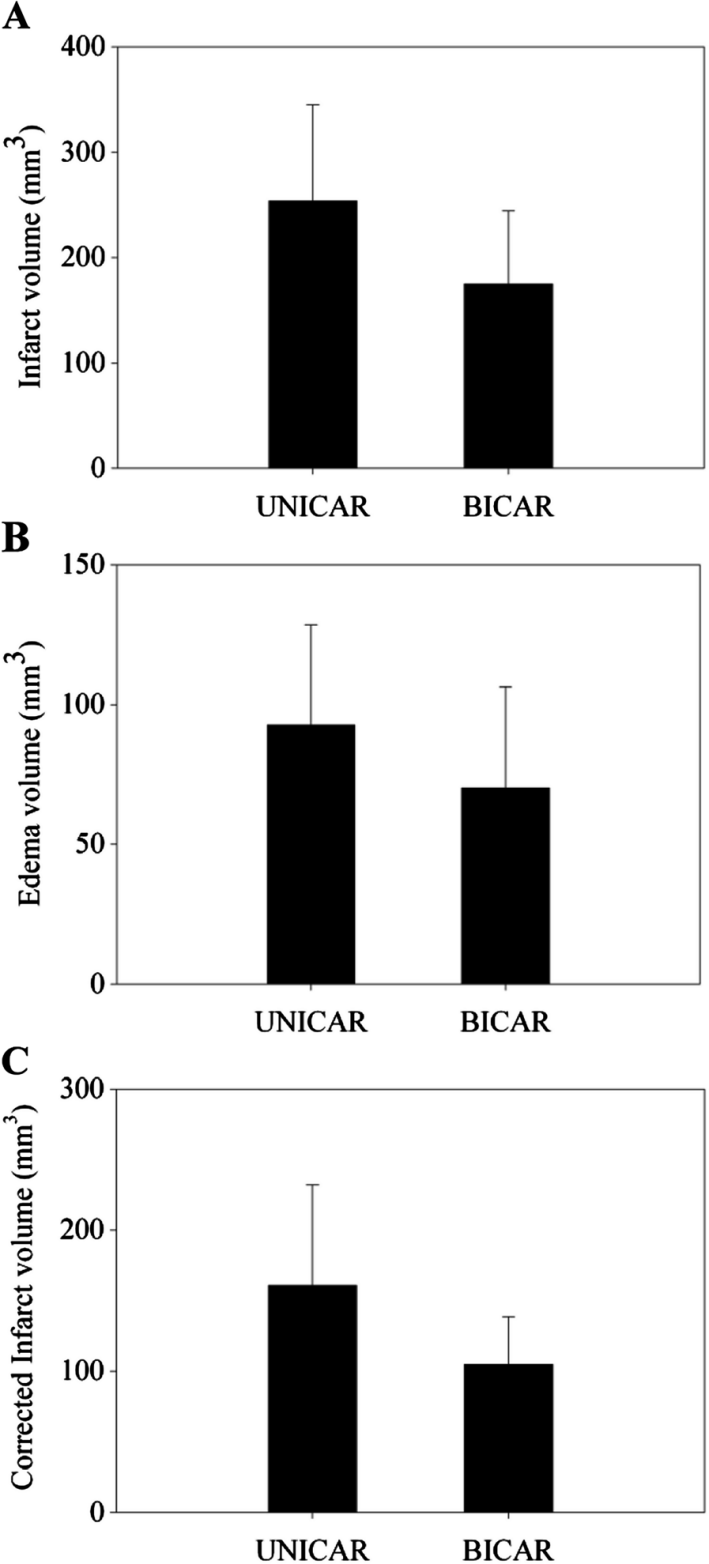
**Comparison of infarct volume (A), edema volume (B), and corrected infarct volume (C) on T2WI between rats receiving unilateral (n = 9) and bilateral carotid artery reperfusion (n = 8). **UNICAR, unilateral carotid artery reperfusion; BICAR, bilateral carotid artery reperfusion.

The comparisons of infarct volume, edema volume, and corrected infarct volume on T2WI are shown in Figure 4. Both infarct volume and edema volume on T2WI were larger in the UNICAR group than in the BICAR group, but these differences were not statistically significant (infarct volume: 253.87 ± 91.32 mm^3^ vs. 174.88 ± 69.47 mm^3^; *P* = 0.066, edema volume: 92.83 ± 35.67 mm^3^ vs. 70.16 ± 36.20 mm^3^; *P* = 0.214, corrected infarct volume: 161.04 ± 71.00 mm^3^ vs. 104.72 ± 34.03 mm^3^; *P* = 0.059). 

### Comparison of DWI lesion volume

The comparisons of DWI lesion volume between two groups are shown in Table 3. There was no statistically significant difference in DWI lesion volume between the UNICAR and BICAR groups after either 60 min (83.75 ± 31.6 mm^3^ vs. 67.52 ± 58.36 mm^3^; *P* > 0.05) or 24 hrs following reperfusion (274.28 ± 80.19 mm^3^ vs. 203.55 ± 65.6 mm^3^; *P* > 0.05). 

**Table 3:** Comparison of DWI lesion volume between the UNICAR and BICAR groups

	**DWI lesion volume (mm**^**3**^**)**	
	**UNICAR (n = 6)**	**BICAR (n = 8)**
1^st^ MRI scan	83.75 ± 31.6	67.52 ± 58.36
2^nd^ MRI scan	274.28 ± 80.19	203.55 ± 65.6

Data are presented as mean ± standard deviation.

1^st^ MRI scan: 60 min following reperfusion;

2^nd^ MRI scan: 24 h following reperfusion.

DWI, diffusion-weighted images; UNICAR, unilateral carotid artery reperfusion; BICAR, bilateral carotid artery reperfusion; MRI, magnetic resonance imaging.

### Comparison of PWI lesion volume

The comparisons of PWI lesion volume between two groups are shown in Table 4. There was no statistically significant difference in PWI lesion volume between the UNICAR and BICAR groups after either 60 min (459.99 ± 213.58 mm^3^ vs. 361.15 ± 366.63 mm^3^; *P* > 0.05) or 24 hrs of reperfusion (466.07 ± 241.14 mm^3^ vs. 428.74 ± 287.8 mm^3^; *P* > 0.05). 

**Table 4:** Comparison of PWI lesion volume between the UNICAR and BICAR groups

	**PWI lesion volume (mm**^**3**^**)**	
	**UNICAR (n = 7)**	**BICAR (n = 8)**
1^st^ MRI scan	459.99 ± 213.58	361.15 ± 366.63
2^nd^ MRI scan	466.07 ± 241.14	428.74 ± 287.8

Data are presented as mean ± standard deviation.

1^st^ MRI scan: 60 min following reperfusion;

2^nd^ MRI scan: 24 h following reperfusion;

PWI, perfusion-weighted images; UNICAR, unilateral carotid artery reperfusion; BICAR, bilateral carotid artery reperfusion; MRI, magnetic resonance imaging.

### Comparison of neurological scores at 24 hours after reperfusion

**Figure 5:**
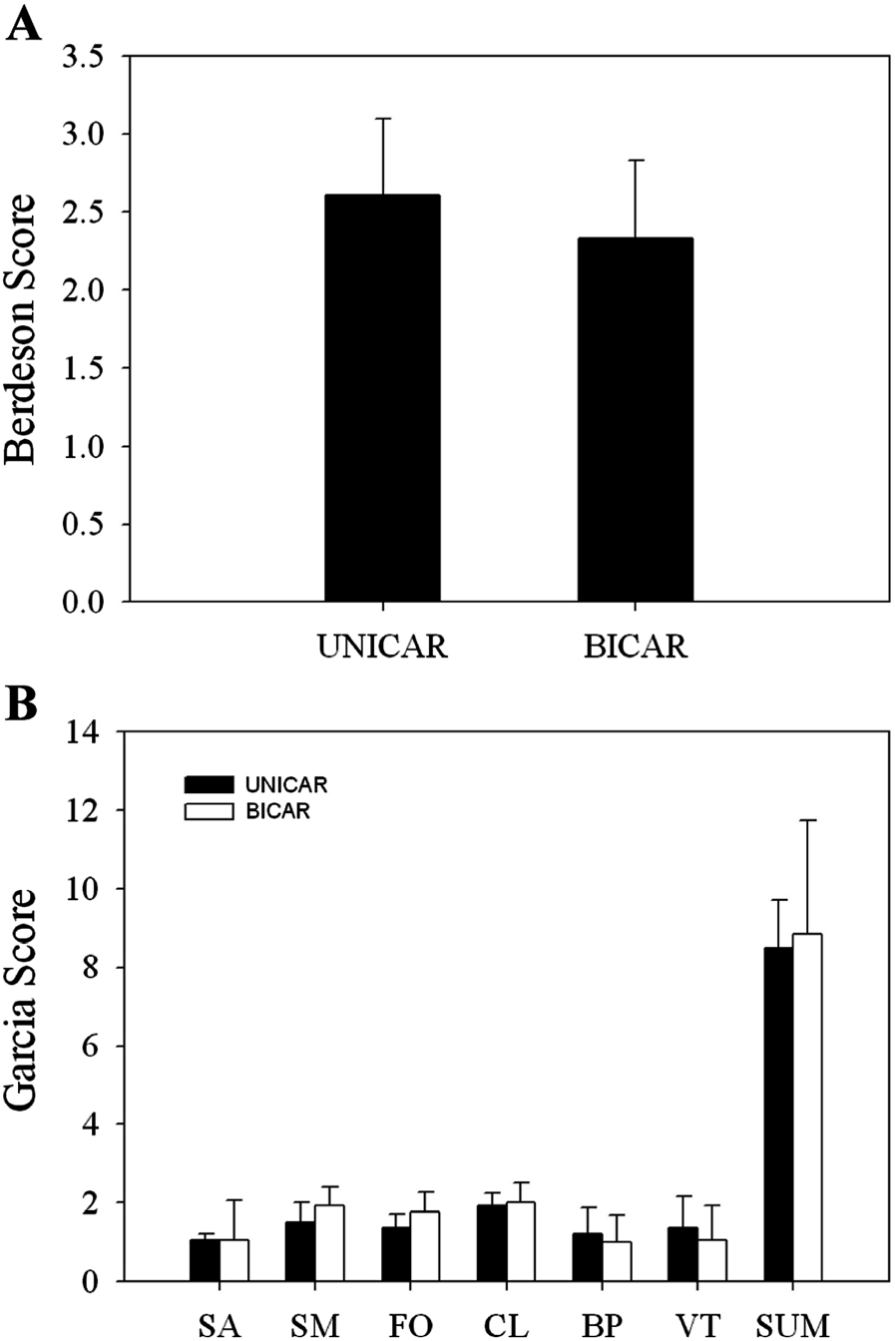
**Comparison of Bederson scale (A) and Garcia scale (B) between unilateral (n = 9) and bilateral carotid artery reperfusion (n = 9) groups. **SA: spontaneous activity; SM: symmetry of movements; FO: forepaw outstretching; CL: climbing; BP: body proprioception; VT: vibrissae touch; SUM: sum of Garcia. UNICAR, unilateral carotid artery reperfusion; BICAR, bilateral carotid artery reperfusion.

When examined using the Bederson scale (Figure 5A), the UNICAR group had higher average scores than the BICAR group, but this difference did not reach statistical significance (2.61 ± 0.49 vs. 2.33 ± 0.50; *P* = 0.249). There were also no significant differences between the UNICAR and BICAR groups in the total Garcia score (8.50 ± 1.20 vs. 8.83 ± 2.9; *P* = 0.757) or in any of the six subscales (Figure 5B). 

### Comparison of mortality rates at 24 hours after reperfusion

A total of 5 rats died. Two rats died of anesthetic overdose and two of subarachnoid hemorrhage; since the causes of deaths were not related to ischemia or the reperfusion methods, these rats were not included in the statistical analysis. Generally, death from these two causes in animal studies of MCAO should according to convention be excluded from the analysis. One rat in the UNICAR group which was still alive 5 hours after reperfusion, had an initial DWI scan and a very large infarction lesion was found. This rat was found dead 24 hours after reperfusion. After its death, we did not find any subarachnoid bleeding and a very large cerebral infarction area could be seen with the naked eye. Therefore, we believe that this rat died of severe cerebral ischemia, which is actually related to the degree of cerebral ischemia or reperfusion method. Therefore, this death should be included in the mortality statistics. Thus, mortality in the UNICAR group was reported as 1/9, and the mortality in the BICAR group was 0. There was no significant difference in mortality between the two groups.

### Comparison of infarct volume, edema volume and corrected infarct volume by TTC staining

**Figure 6:**
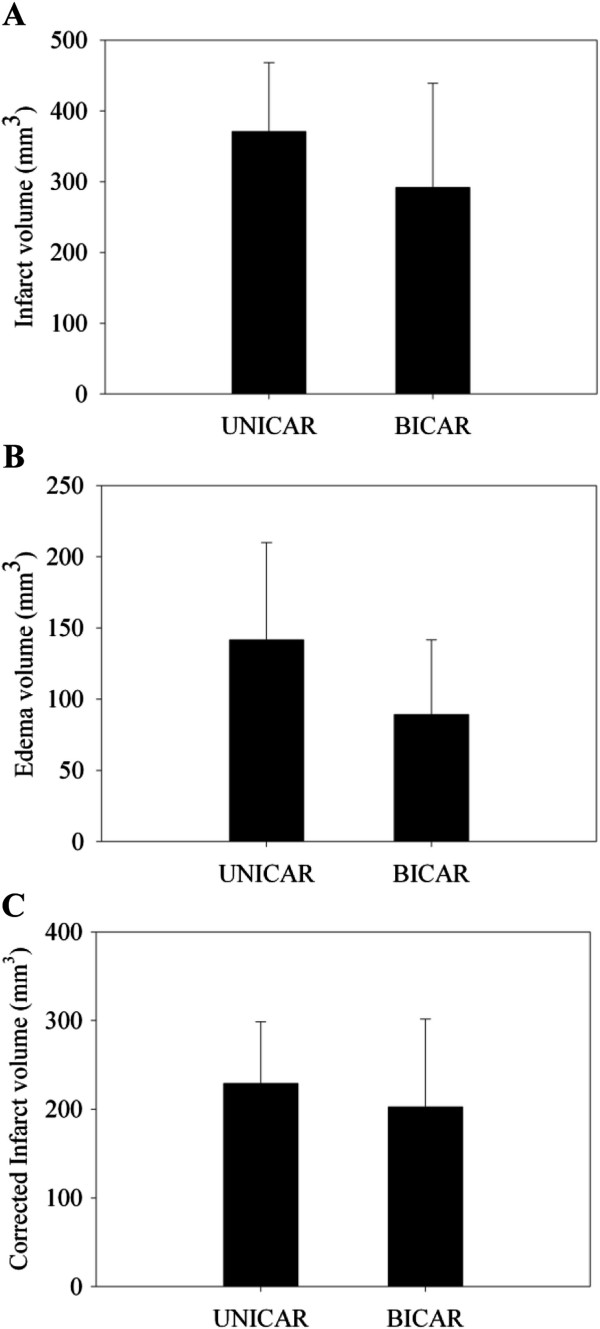
**Comparison of infarct volume (A), edema volume (B), and corrected infarct volume (C) by TTC staining between rats receiving unilateral (n = 9) and bilateral cerebral artery reperfusion (n = 9). **UNICAR, unilateral carotid artery reperfusion; BICAR, bilateral carotid artery reperfusion.

The comparisons of infarct volume, edema volume, and corrected infarct volume by TTC staining are shown in Figure 6. Both infarct volume and edema volume by TTC staining in the UNICAR group did not differ significantly from the BICAR group (infarct volume: 370.78 ± 97.73 mm^3^ vs. 291.69 ± 147.36 mm^3^; *P* = 0.198, edema volume: 141.60 ± 68.37 mm^3^ vs. 89.23 ± 52.32 mm^3^; *P* = 0.087, corrected infarct volume: 229.18 ± 69.52 mm^3^ vs. 202.46 ± 99.12 mm^3^; *P* = 0.517). 

## Discussion

In this study we found that counter to our original hypothesis, there were no statistically significant differences between the UNICAR and BICAR groups in all parameters observed. We initially hypothesized that there would be higher blood flow obtained by using bilateral reperfusion, which would result in better physiological and behavioral outcomes. However, no such differences were found. These similarities were unexpected as changes in variables such as age [3] and strain [2] of the animal, filament type [5], and ischemia duration [4,26] result in different outcomes in the effects of MCAO, so it was thought that major differences in reperfusion technique would also result in differences in both histological and functional neurological outcomes.

Infarct size is positively correlated with the duration of transient ischemia [4,26]. As we expected BICAR to result in faster and more widespread increases in blood flow, this should have resulted in smaller infarct size, which did not occur. A possible explanation for this would be that the cerebral vascular system of rats is able to compensate for the loss in blood supply from the ipsilateral carotid artery during the reperfusion period. Anatomically, this could be the result of the vertebral arteries (via posterior communicating arteries and pial collateral vessels) and contralateral carotid artery (via anterior carotid artery and pial collateral vessels) supplying the ischemic cerebral hemisphere during reperfusion [7,27]. Interestingly, while duration of ischemia correlates with infarct size, reperfusion has also been found to produce brain damage. Deleterious biochemical processes, particularly the formation of ubiquitin aggregates, during reperfusion after ischemia have been shown to antagonize the beneficial effects of oxygen and glucose supply reinstatement [28]. In the Aronowski et al. study, rats that underwent unilateral MCA/CCA occlusion lasting 120–300 min exhibited significantly greater infarct volume than rats receiving permanent occlusion [29]. Accumulation of ubiquinated protein aggregates after cerebral ischemia has been thought to cause neuronal degeneration [30]. In addition, results from a recent study suggest that reperfusion rather than ischemia is the triggering factor for ubiquination [29]. The results from the current study may in line with the theory that both protective and deleterious events are associated with reperfusion after ischemia.

The primary limitation of our study was that we did not observe long-term neurobehavioral outcomes of rats with ischemia/reperfusion, only a single 90 min duration of ischemia was performed. Also, as cerebral ischemia can have effects on various physiological functions, it would be impossible to account for all of them in the current study. Future studies using longer ischemia and survival and, as well as examining other physiological parameters will be necessary to determine this. Another limitation is that we used the 3.0T MRI scanner that is used in clinical practice to scan the rats. Although the scanning coils were specialized coils for rats, using an MRI scanner designed for clinical use may produce some errors. In the future, we plan to perform post-ischemic cerebral perfusion studies in rats using an MRI instrument designed for animals with a higher field (>7T), which may reduce the variability.

## Conclusions

We conclude that despite our original hypotheses, the UNICAR and BICAR techniques resulted in similar neurophysiological and histological outcomes at 24 hrs after ischemia/reperfusion, the time commonly used for observation of the neuroprotective effects of various compounds in male Sprague Dawley rats. These similarities of effects were found across all parameters, including infarct volume, the effects on the ischemic tissue itself, and locomotor deficits commonly associated with unilateral cerebral ischemia. These results suggest that while other variables involved in induction of ischemia will influence outcomes, the type of reperfusion method may not. This finding is potentially of great benefit to the scientific community as it allows comparison of studies using different reperfusion techniques without worrying about the introduction of confounding variables and it also allows experimenters to choose which reperfusion technique to use based on practical matters instead of difference in effects. However, additional studies using longer periods of assessment than 24 h after reperfusion are needed to evaluate possible differences in neurological outcome between the two methods.

## Competing interests

The authors declare to have no competing interests.

## Authors’ contributions

JRL contributed to study concepts, study design, definition of intellectual content, literature research, experimental studies, data acquisition, data analysis, statistical analysis, manuscript preparation and drafting of the manuscript. URJ contributed to definition of intellectual content, literature research, data analysis, statistical analysis, manuscript editing and manuscript review. JJZ, FS, XYF and XLS contributed to experimental studies and data acquisition. GD contributed to manuscript review and supervision. OJ is the guarantor of integrity of the entire study, supervision. TH contributed to manuscript review. JM contributed to experimental studies and data acquisition. YZ contributed to experimental studies and data analysis. CE contributed to study concepts, study design, definition of intellectual content, literature research, manuscript editing, manuscript review and supervision. All authors read and approved the final manuscript.

## Authors’ information

Shared authorship: Jian-Ren Liu, Ulf R. Jensen-Kondering.
